# Outline of a Lecture

**Published:** 1877-12

**Authors:** James B. Hodgkin


### ARTICLE II.

### *Outline of a Lecture, in the Preliminary Course, at the Baltimore College of Dental Surgery.*

BY PROF. J. B. HODGKIN.

GENTLEMEN :—I take it that you who are assembled here in this lecture room this afternoon, are so thoroughly in earnest in your desire to learn, as to require no arguments addressed to your understandings or stimulants to effort from myself or from others, in that direction. I am quite sure that not one who has matriculated for this winter's course of lectures, is disposed to do any other thing than to earnestly grapple the scientific and practical truths which may be presented to his mind, and with a firm determination to master them ; that they are not anxious about anything so much as that they shall get the utmost good of their coming here, and shall carry away with them all of Dentistry that they can possibly learn. And taking this for granted, I feel that these facts gives me an advantage in speaking to you, which I can hardly underrate. For if it be true that any enthusiasm of the lecturer is communicated to the hearer, it is even more true, in a lecture room at least, that a class brimming over with zeal, and yearning after the truths of science, may incite the lecturer to efforts which the presence of a listless audience could never inspire. I feel then that I need only bespeak your attention in order to obtain not only a respectful hearing, but a cordial, unreserved confidence.

It may seem strange to you, that I use this last word, *confidence*, but constant contact with students has led me to conclude that such is the perversity of human nature and such the reliance of young men on their own judgment, and such often the undue influence of their previous training, that they in occasional instances seem to conclude that they know enough before coming here, and that their presence in an institution of this kind is only in deference to a preverted and erroneous public opinion which requires such presence and such tuition. The "smart young man" of a class, who knows everything beforehand, is not a pleasant subject for teaching, and often he finds that his previous knowledge is of little practical use, I had almost said it is well when he does find it out. Indeed if I could have it so,—if I could have the direction of your previous mental training, I am not at all sure that I should select a "preceptor" for the purpose, but should prefer you to take your "course of lectures" *first*, and your pupilage afterwards. It is quite certain the individuality of eight men is collectively less likely to influence you injuriously than the individuality of one, whose methods of practice, assuming that he allows you a full insight into them, are likely to be more or less influenced by his needs.

As we face each other to-day, I feel more than ever the importance of a condition I have previously hinted at,—a condition of cordial receptivity on your part. I tell now in the outstart, that you will lose little and gain much by throwing away all spirit of cavil and doubt, and accepting as the truth the utterances from this desk. It will save you much discomfort and uncertainty if you willingly and heartily take what is taught you here as the *truth*, and assume that those who make utterances seemingly at variance with your preconceived notions have authority for their statements; that they have investigated these things and bring you as the results of their research the truths of science. And indeed it is obvious to you that this is the end and aim of establishing a Dental School,—that the labor of individual investigation may be crystallized, and the work of solitary thinkers and discoverers, scattered here and there, collected and utilized, that you may enjoy in full the results of these labors.

But I pass this part of the subject, and briefly call your attention to a few thoughts in connection with our work for the winter. And in respect to the nature of it, it is well to say that it differs so materially from the development required in any other profession, as to make ours a peculiar occupation, calling for peculiar talents as well as special training. Our work divides itself into—

Head Work.

Hand Work.

Now I have yet to see the student who did not at the first underrate the importance of the first division I make, and overrate the importance of the last; and it is on this account that I wish to speak of it at the outset of our winter's study. I have said that our occupation is a peculiar one, calling for special talents and gifts. The lawyer, the physician, the journalist, the merchant perhaps, needs mental training only. The barrister may be so clumsy with his fingers as scarcely to be able to tie up his bundle of papers, but this awkwardness does not interfere with his efficiency at the bar. The physician, now that the days of the lancet are numbered, need have no other use for his hands—unless he practices surgery, which is fast passing into the hands of the specialist—than will serve to tie his horse, or write a prescription; and so with others. But the dentist is nothing unless his tactual dexterity is cultivated to a sense more delicate than that of vision itself; and he must combine and unite with this delicacy of touch, strength, endurance, quickness of perception, a precision of execution little less than is called for in the highest developments of art in any direction. So, students, early recognising this as so important, are anxious to let no opportunity slip of cultivating their fingers, and are too apt to neglect the other and more important pursuit, the acquisition of knowledge.

I am quite sure that you will agree with me ten years from now, on this subject, although it may seem a hard thing to accept at present. But if we consider the matter a few moments, we will see that in order to be able to *do*, we must know what to do; before we can execute, we must conceive; before we can cure disease we must know its origin, its manner of progress, its probable conclusion or result. "He that follows must always be behind," is an old maxim, and if you only learn routine practice, failing to grasp the great fundamental principles which underlie science,—and only hard study will enable you to do this—you will find yourselves very helpless when, working for yourselves, you meet with cases which depart from the route you mechanically pursued. If you do not learn to think for yourselves, to diagnose with precision, your work as dental surgeons must be uncertain, and your triumphs over the difficulties inherent in your profession less numerous than your failures; you will surely retrograde following a beaten path of practice; you cannot but advance if you lay firm and broad the foundation of your profession in your minds.

Had we the time,—but how little could we learn in this way in the few short months we are together here!—we would prefer going to the fountain head of all instruction, and study out from nature for ourselves the whole problem of dental practice, with its correlated branches. But the whirl of events push us, and we must get our knowledge in any and every way that it will come to us, taking advantage of all the adventitious aids we may be able to obtain, and taking in first and analyzing afterwards the theories we hear and read; for mental digestion is like that of the ruminant animals, and it is not diffieult to put away facts for reconsideration.

Further back I stated that no one can execute that which he cannot conceive. The mind must be the guide of the eye and of the hand; and I take occasion to say further, that no man is so safe as he who has a good theory to work by. The men who decry *theoretical* knowledge, and who vaunt themselves as superior on account of their *practical* skill, little realize how entirely they are dependent on the very theories they sneer at, little see that but for the *thinkers* they would have nothing to practice. It is not on account of their theories that these fail in the execution of their schemes; it is rather due to the fact that the power to conceive and to execute are seldom equally resident in one individual; but you readily see that the theorist must always precede the hand worker, in this as well as other professions. That the combination of the two qualities is rare, is not due to the fact that the theorists' mental powers were developed at the expense of his physical; there is a law of compensation in nature, but it does not hold good here.

I would like to encourage you in this pursuit of knowledge if it were only for the sake of knowing, to animate you to enthusiasm by the examples of the illustrious men of your to be profession, showing you that no knowledge comes amiss to its fortunate possessor. But I will consider this branch of the subject in a different aspect, and on a lower plane, and show you how in a merely selfish and mercenary point of view it is important that you acquire not only a knowledge of the facts directly related to your future practice, but those remotely related, and more directly related to the practice of medicine, of which we consider our calling a branch. It is very certain to my mind,—long observation has made it perfectly clear,—that just in proportion as you prove yourself the peer of your medical associates, just in that proportion will they respect and esteem you; just in proportion as you show that you are possessed of knowledge about *their* work, they will look on you with deference, and consult your opinion with confidence. One need not indeed make a parade of his knowledge, and display his weakness of judgment as he shows his acquirements, but he can and should cultivate the society of medical men, and be able to discuss with positive knowledge, questions directly or remotely related to the dental organs;—and you will see further on in these lectures that this is a more complex subject than you dreamed of;—and it is probable if you are sagacious you can show your medical friend that you can help him in many an obscure diagnosis. So that, looking at this matter in only this selfish way, we must see that the broader our cultivation, the more certain our success, the more positive our gain; and success and gain are strong inducements to most men.

But there is a higher aim than this of which for a moment I speak before I conclude. We are as I said, members of the healing art, brothers of the great fraternity whose noble function it is to assuage the pangs of suffering humanity. It is our noble privilege,—and can there be a more exalted calling?—to relieve the suffering sons and daughters of men of suffering, all the more formidable on account of its disastrous consequences, taking into its injurious embrace almost every organ of the system. We may not be successful in making fortunes out of our profession—the talents that give success in that direction, would give success in any pursuit in life; we certainly will not be successful in avoiding hard work. Our calling is one of the most laborious of the professions. But here on this broad platform of the HEALER, here on this elevated field where we stand by the side of and feel ourselves the peer and equal of the physician, we may and should find scope for our highest power and feel that in the exercise of the accumulated knowledge and skill, we are compensated in a way which no fee or reward can bring us. Doing our full duty to our patients is itself a most honorable, dignified, ennobling pursuit. See to it that you do this fully.

"What is our duty," may be hereafter considered.

